# Hierarchical micro-/mesoporous zeolite microspheres prepared by colloidal assembly of zeolite nanoparticles

**DOI:** 10.1039/d0ra07394f

**Published:** 2020-10-02

**Authors:** Walter Rosas-Arbelaez, Andreas J. Fijneman, Heiner Friedrich, Anders E. C. Palmqvist

**Affiliations:** Department of Chemistry and Chemical Engineering, Applied Chemistry, Chalmers University of Technology SE-41296 Gothenburg Sweden arbelaez@chalmers.se; Laboratory of Physical Chemistry, Center for Multiscale Electron Microscopy, Department of Chemical Engineering and Chemistry Eindhoven, University of Technology Groene Loper 5 Eindhoven 5612 AE The Netherlands anders.palmqvist@chalmers.se

## Abstract

A novel template-free colloidal assembly method that combines colloidal zeolite (silicalite-1) suspensions in a water-in-oil emulsion with an evaporation-induced assembly process has been developed for preparing hierarchical micro-/mesoporous zeolite microspheres (MZMs). Such particles have an interconnected mesoporosity and large mesopore diameters (25–40 nm) combined with 5.5 Å diameter micropores of the zeolite nanoparticles. The method developed has the advantages of employing mild synthesis conditions, a short preparation time, and not requiring the use of a mesoporogen template or post-treatment methods. The method provides a new range of micro-/mesoporous zeolites with tunable mesoporosity dictated by the size of the zeolite nanoparticles. It also offers the possibility of combining several zeolite particle sizes or optionally adding amorphous silica nanoparticles to tune the mesopore size distribution further. It should be generally applicable to other types of colloidal zeolite suspensions (*e.g.* ZSM-5, zeolite A, beta) and represents a new route amenable for cost-effective scale-up.

## Abstract 2

Evaporation-driven colloidal assembly of silicalite-1 nanoparticles into well-defined micro-sized spheres at low temperature and preparation times.

## Graphical abstract

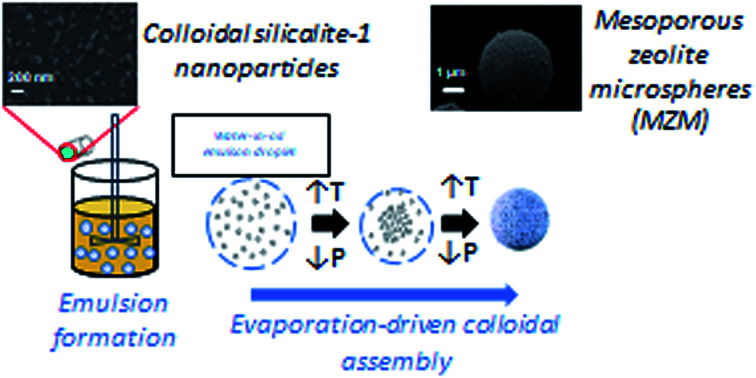

## Introduction

Zeolites have been extensively studied and used for decades as catalysts, sorbents and ion exchange materials.^1^ They are crystalline microporous (<20 Å pore diameter) aluminosilicates and contain ordered arrangements of interconnected channels formed from silica and alumina tetrahedra, enabling a molecular sieving effect for separation of a variety of molecules (*e.g.* CO_2_).^2,3^ Ion-exchanged zeolites are one of the most frequently used catalysts in industry. In the proton exchanged form they are used for oil refining and gasoline production due to their strong acidity as well as size and shape-selective properties.^4,5^ In transition metal ion-exchanged form, they are used in exhaust gas catalysis for lean NO_*x*_ reduction due to their selective redox chemical properties.^6,7^ In addition, zeolites have also shown potential in water purification, large biomolecule separation, and removal of radioactive contaminants.^8–10^

An important aspect to consider in the design of zeolite-based catalysts is the intracrystalline diffusion of gas molecules in narrow micropores, since it may restrict the performance of zeolites in adsorption and desorption processes, central to catalytic conversion.^11–13^ Limitations in the diffusion not only reduce the catalytic performance but also affect the selectivity and durability of the catalyst.^14^ Some of these limitations can be overcome either by ultra-large pore zeolites or with short diffusion paths as those of zeolite nanoparticles.^15–19^ Reduction of the zeolite particle size improves the volumetric diffusion rate into and out from the zeolite and thereby also the catalytic conversion rate while maintaining the shape selectivity of the zeolite. In addition, the reduction of zeolite particle size increases the outer specific surface area of the material, which for reactions involving bulky molecules may be favorable.^20^ However, for catalytic and separation applications, nanozeolites are difficult to recycle after use. In addition, packing nanozeolites in bed reactors or chromatographic columns can create high pressure drops.^21^ A promising complementary strategy to tackle diffusion limitations in zeolites is to incorporate either meso- or macropores in the materials. This topic has received a lot of interest, since the combination of properties from different types of porosities could potentially enhance the overall mass transport of reagents and products to and from the catalytically active sites.^11,22–24^

During the last two decades, several researchers have thus focused on developing strategies to form hierarchical porous zeolites. The synthesis protocols can be divided into three different categories of materials: micro-/meso-/macroporous zeolites, micro-/macroporous zeolites and meso-/macroporous zeolites.^11^ From the perspective of the catalytic and separation applications, a micro-/mesoporous material with well-defined morphology and high catalytic activity is preferable, since mesopores have a desired pore size domain for improved mass transport as well as the well-defined morphology with uniform size (spheres in micro-size range) influences the rapid adsorption and desorption of the molecules.^13,25,26^ Various micro-/mesoporous zeolite synthesis approaches have been successfully developed, but the optimization of interconnectivity between the different pores as well as the importance of simple and scalable methods, still represent major challenges.

Micro-/mesoporous zeolite structures have been prepared using various strategies that can be divided in three groups: post treatments, soft templates and solid templates.^11,27^ Although, many of these approaches have successfully provided micro-/mesoporous structured zeolites, in some cases there have been significant drawbacks. These include reduction of the crystallinity and acidity, isolated cavities, limitations in accessibility and mass transfer for bulky molecules due to small mesopore sizes and reduction in the catalytic activity.^11,27–31^ Moreover, these approaches are often expensive, quite limited by available template sizes, slow, require high crystallization temperatures and lack incorporation of alumina tetrahedrons, which are critical for catalytic activity.

One group of synthesis strategies that has been less investigated but overcomes some of the aforementioned issues is the indirect templating approach.^27,32^ The indirect templating methods do not use any type of mesopore template, which has a significant effect on the synthesis cost. In addition, the indirect templating strategies can lead to higher mesopore sizes and pore volumes. Steam-Assisted Crystallization (SAC) has been used to prepare mesopore zeolite beta and MFI-type zeolites, obtaining mesopore size distributions up to 60 nm and pore volumes up to 0.9 ml g^−1^.^33–35^ Another method is the nanofusion, at which nanozeolites are fused into hierarchical zeolite aggregates under drying and calcination steps.^36^ Mesoporous zeolite beta was prepared by nanofusion of zeolite crystals of 20–40 nm, forming mesopores up to 29 nm in size with surface areas between 656 and 766 m^2^ g^−1^. However, some of these methods still have some drawbacks: high crystallization temperatures, long preparation times, low control of the mesopore size and non-defined morphology of the particles.

Recently, mesoporous silica microspheres were prepared by colloidal assembly of silica nanoparticles without the use of mesopore templates and post-treatment steps.^37^ This strategy can successfully provide tuned and controlled pore size and pore volume by means of two approaches: evaporation-driven and gelation-driven colloidal assembly. The evaporation-driven colloidal assembly is a method used to produce large close-packed particles sometimes called supraparticles, supraballs or supracolloids.^38–40^ In this method, random suspended nanoparticles within emulsion droplets are driven into close packing by gradually removing the carrier liquid until the colloidal particles assemble into these supraparticles.^37,41–43^ While the carrier liquid is removed by evaporation, the droplets shrink, and the concentration of the contained nanoparticles within the droplets increases until there is no more free space between the nanoparticles, and they assemble into a dense-packed sphere.^44^

In this work, we present a simple and efficient evaporation-driven template-free colloidal assembly procedure for the preparation of hierarchical micro-/mesoporous zeolite microspheres (MZMs). We use a sol of discrete colloidal zeolite particles as building blocks and as the aqueous part of a water-in-oil (W/O) emulsion. By judicious choice of process parameters and of zeolite particle size and composition of the aqueous phase, the pore size, pore volume and structure of the microsphere could be controlled. The assembly process can be readily scaled up and is easily induced by evaporating the water from the droplets under vacuum conditions resulting in well-defined micrometer-sized spheres.

## Experimental part

### Materials

The silica source used for the silicalite-1 synthesis was tetraethyl orthosilicate (TEOS, > 98%, Sigma-Aldrich) and the alkali was tetrapropylammonium hydroxide (TPAOH, 1.0 M aqueous solution in water, Sigma-Aldrich). Phenethyl alcohol (PEA, ≥99%, Sigma-Aldrich) used as the oil phase and hydroxypropyl cellulose (HPC, average *M*_w_ = 100 000 g mol^−1^, Sigma-Aldrich) as a stabilizer and emulsifier were purchased from Sigma Aldrich. The colloidal silica sol used in this work was provided by Nouryon Pulp and Performance Chemicals AB, Sweden and consists of a suspension (sol) of colloidal silica nanoparticles of 25 ± 5.8 nm average diameter and 15 wt% of SiO_2._ The sol was ammonium stabilized (pH of 8–10). The water used in this work was deionized by a Milli-Q Advantage A10 system (Merck Millipore) and had an electrical resistivity of 18.2 MΩ cm at 25 °C.

### Preparation of silicalite-1 sol

Colloidal silicalite-1 particles were prepared following a procedure reported by Persson (now Palmqvist) *et al.*^45^ In short, 4.5 g of tetraethyl orthosilicate (TEOS) was added to 7.2 g of tetrapropylammonium hydroxide (1 M TPAOH) solution with magnetic stirring in a polypropylene bottle for 24 h at room temperature. The clear solution was then heated at 98 °C for 24 h. The colloidal zeolite particles were separated by centrifuging the suspension in a Labofuge 200 (Heraeus Sepatech) at 5600 rpm for 1 h. The supernatant was carefully decanted, and the solid residue was redispersed in water by sonication for 1 h, after which the sample was once more centrifuged and redispersed in water. The colloidal silicalite-1-sol concentration was adjusted to 10 wt%.

### Preparation of MZM particles

The new method of evaporation-induced assembly of the colloidal zeolite particles was used to form micro-/mesoporous zeolites from the colloidal silicalite-1 sol. A 3 wt% aqueous solution of hydroxypropyl cellulose (HPC, *M*_w_ ∼100 000, Sigma-Aldrich) was used as emulsifier and stabilizing agent. It was prepared by dispersing 3 g of HPC under vigorous stirring in pre-heated Milli-Q water (97 g) at 60 °C in a reflux setup to avoid water evaporation. After one hour the solution was cooled down gradually to room temperature and was kept under continuous stirring for up to 24 h. After the HPC was fully dissolved, the solution was filtered twice over filter paper (pore size 5 μm, Munktell) and stored in a plastic bottle. An oil phase was prepared by mixing 50 g phenethyl alcohol (PEA, Sigma Aldrich) and 4.2 g of the 3 wt% HPC solution in a beaker at room temperature and stirring at 300 RPM for one hour with an overhead mixer (IKA Yellow line OST Basic), at which point the mixture was fully transparent. 10 g colloidal silicalite-1 sol (10 wt%) was then added under constant stirring (350 RPM) to form a water-in-oil emulsion with a white/milky appearance. After 60 minutes stirring, the mixture was poured in a round bottom flask and attached to a rotary evaporator fitted with a water bath (KNF RC-900) and rotated at the temperature of 65 °C and at a reduced pressure of 160 mbar. Rotary evaporation was continued for 90 minutes, at which point most of the water was removed, resulting in an almost totally clear/transparent mixture. The temperature was then increased to 85 °C for 30 minutes more, before being allowed to cool to room temperature. The resulting MZM product was separated from the mixture by filtration over a Pyrex glass filter, dried at 90 °C for 16 h and then calcined in air at 650 °C for 4 h to remove the organic template from the zeolite pores.

### Characterization

The morphology of the colloidal silicalite-1 particles and the MZM particles were examined by means of scanning electron microscopy (SEM) on a Carl Zeiss ULTRA 55 microscope with an acceleration voltage between 1.5 and 3 kV. The samples were placed on a carbon paper and coated with a thin palladium layer. XRD patterns were measured using a Siemens D-5000 X-ray diffractometer. The surface and pore properties of the calcined colloidal silicalite-1 particles and the MZM were determined by N_2_-physisorption at 77 K using a TriStar 3000 instrument from Micromeritics. The samples were degassed at 503 K for 12 h in dry N_2_ prior to the measurement. The specific surface area was obtained using the Brunauer–Emmett–Teller (BET) theory and pore size distributions using the Barrett–Joyner–Halenda (BJH) model. The total pore volume was estimated at *p*/*p*_0_ of 0.99.

## Results and discussion

**Scheme 1:**
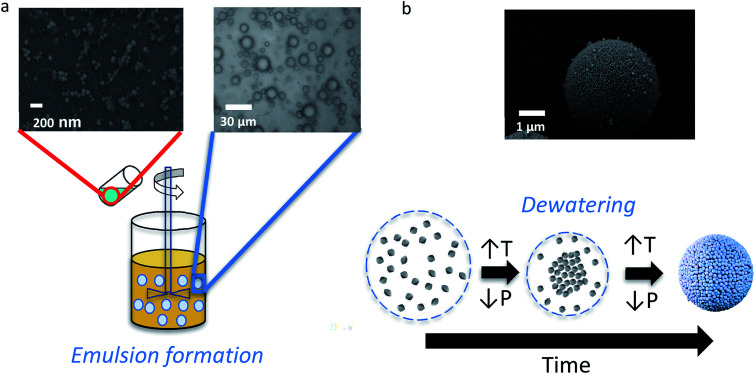
General synthesis of mesoporous zeolite microparticles (MZMs) prepared by colloidal assembly. (a) Formation of water-in-oil emulsion with colloidal silicalite-1 particles, (b) evaporation of emulsion and formation of hierarchical micro-/mesoporous zeolite spheres *via* assembly of the colloidal silicalite-1 particles.

The design of the synthesis process for perfectly spherical micro-/mesoporous zeolite microspheres (MZMs) was inspired by previously published literature for mesoporous silica microspheres.^37,44^ The synthesis process is illustrated in Scheme 1 and based on the use of previously prepared discrete zeolite nanoparticles as building blocks.

The formation of hierarchical MZMs can be described as follows. First, a W/O emulsion was prepared by adding a colloidal zeolite suspension (10 wt% in water) to a mixture of an oil phase and an emulsifier under stirring. At this stage, the discrete colloidal zeolite particles are randomly distributed throughout the emulsion droplets. After the emulsification process, the formation of the MZMs is induced by evaporation of water from the emulsion under mild temperature (65 °C) and reduced pressure conditions (160 mbar), which leads to a shrinkage of the droplets and a homogeneous aggregation and gelation of the colloidal zeolite particles. Similar to the case of using amorphous silica particles in the formation of mesoporous silica microspheres (MSMs), the gelation presumably occurs due to formation of siloxane bridges between the silicalite-1 particles through the reaction of isolated deprotonated silanol surface groups.^37^

**Fig. 1:**
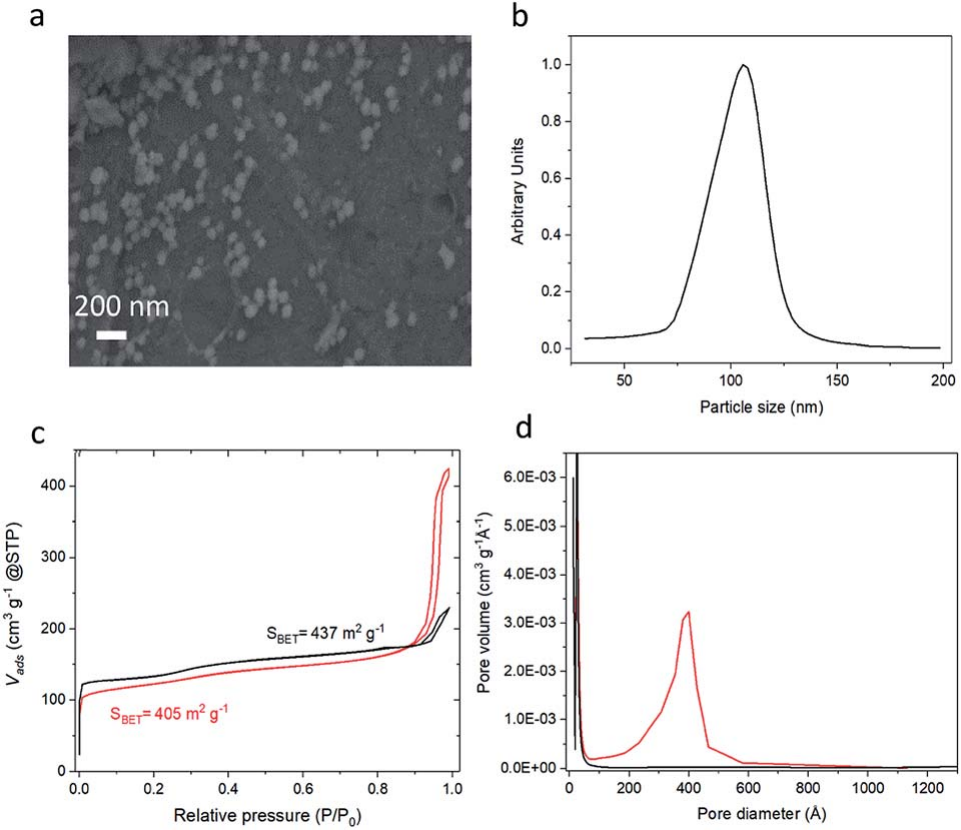
Characterization of silicalite-1 MZMs and of colloidal silicalite-1 particles used to prepare them. (a) SEM image of the colloidal silicalite-1 particles, (b) particle-size distribution of the colloidal silicalite-1 particles as determined by analytical ultracentrifugation (AUC), (c) N_2_ adsorption/desorption isotherms of colloidal silicalite-1 particles and silicalite-1 MZMs, and (d) N_2_ adsorption based pore size distribution of colloidal silicalite-1 particles and silicalite-1 MZMs. (Black line: colloidal silicalite-1 particles; red line: silicalite-1 MZMs).

To demonstrate the method, colloidal zeolite silicalite-1 particles were used to prepare MZM particles. Prior to the preparation of the MZMs, colloidal silicalite-1 particles were prepared following a previously developed procedure.^45^ Prior to use, the colloidal suspension was purified through two cycles of centrifugation and redispersion. A SEM image of the colloidal silicalite-1 particles used as building blocks is shown in Fig. 1a. The image shows that the particles have a quasi-spherical shape with an average particle diameter of 105 nm. A similar average particle size was obtained by analytical ultracentrifugation (AUC) shown in Fig. 1b. From Fig. 1b a coefficient of variation of around 10% was calculated, indicating a quasi-monodisperse distribution of the silicalite-1 particles.^46^ The silicalite-1 particles were also characterized by means of N_2_ adsorption/desorption analysis, and we observe a typical isotherm of type I (see Fig. 1c), characteristic of microporous materials.^47^

When mixing the aqueous silicalite-1 nanoparticles sol with an oil phase consisting of a mixture of phenethyl alcohol and hydroxypropyl cellulose (HPC) as emulsifier a W/O emulsion is formed as shown with optical microscopy in Scheme 1, illustrating the aqueous droplets embedded in the oil phase. The droplet size distribution is relatively broad or polydisperse with most of the droplets in the range between 2 and 25 micrometers, which is within the range of typical values for droplets in macroemulsions.^48^ The subsequent increase in temperature and reduction of the pressure leads to dewatering of the droplets and the associated assembly of the colloidal particles, resulting in the formation of spherical mesoporous zeolite particles.

The N_2_ physisorption isotherm of the silicalite-1 MZM in Fig. 1c shows a type IV isotherm, which is characteristic for mesoporous materials.^47,49^ From the N_2_ physisorption analysis, small decreases in the specific surface area and the micropore volume were observed between the colloidal zeolite nanoparticles and the MZM, from 437 m^2^ g^−1^ to 405 m^2^ g^−1^ and 0.145 to 0.116 cm^3^ g^−1^, respectively. This is attributed to the assembly of the colloidal zeolite particles into the colloidal supraparticles, which is expected to result in such losses. However, a significant increase in the total pore volume from 0.20 cm^3^ g^−1^ of the colloidal zeolites to 0.64 cm^3^ g^−1^ of the MZM was observed (see Table 1), due to the formation of the mesopores. The mesoporosity shows an average pore size peak at 38 nm (see Fig. 1d). The homogeneous aggregation of zeolite nanoparticles thus creates a homogeneously distributed interconnected mesoporosity with a high pore volume roughly on the order of 1/3 the diameter of the zeolite particle size. This is typical for aggregated spheres prepared by an evaporation-driven approach.^37^ We emphasize here that this approach does not use organic or inorganic templates to form these mesopores, which instead are formed solely by the evaporation-induced colloidal particle assembly process.

**Table 1:** Textural parameters of colloidal silicalite-1 particles, silicalite-1 MZM and MZSM particles

Sample	*S*_BET_ (m^2^ g^−1^)	*V*_mic_ (cm^3^ g^−1^)	*V*_total_ (cm^3^ g^−1^)
Colloidal silicalite-1 particles	437	0.145	0.20
MZM particles	405	0.116	0.64
MZSM particles	240	0.055	0.41

Our developed MZMs exhibit one of the highest pore volumes and certainly the lowest preparation time and temperature reported for synthesis of mesoporous zeolites when compared with other reported mesoporogen-free methods, as shown in Table 2.

**Table 2:** Comparison of total pore volume, preparation temperature and time of secondary template-free methods for mesoporous zeolites

Method	Type of zeolite	*V*_total_ (cm^3^ g^−1^)	Preparation temperature^a^ (K)	Preparation time^b^ (days)	Reference
Evaporation-driven colloidal assembly	Silicalite-1	0.64	358	0.5	This work
Nanocrystal aggregation	ZSM-5	0.25	408	1–4	Wang *et al.*^50^
Self-pillared zeolite nanosheets	ZSM-5	0.7	388	1–3	Zhang *et al.*^51^
Steam assisted conversion (SAC)	ZSM-5	0.22	443	3	Jia *et al.*^52^
Rod-like nanocrystal assembly	ZSM-5	0.42	423	3	Zhang *et al.*^53^
Crystallization amorphous gel	Silicalite-1	0.11*	370	2–8	Naik *et al.*^54^

a Temperature condition at which the method was carried out.

b Time condition of the used method. Excluding pre-synthesis step, ageing or post-treatment times.

**Fig. 2:**
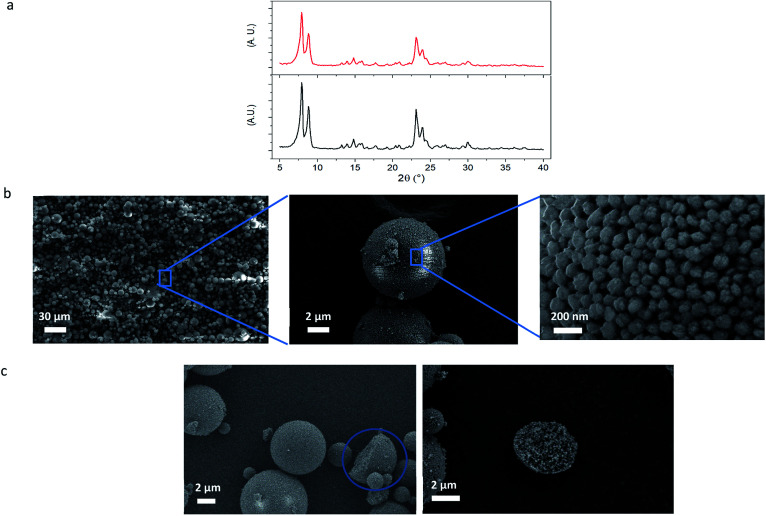
Characterization of MZM particles. (a) X-ray diffraction pattern for colloidal silicalite-1 (black line) and silicalite-1 MZM (red line), (b) SEM images of the obtained silicalite-1 MZM spherical particles, and (c) cracked silicalite-1 MZM particles.

Powder X-ray diffraction (XRD) analysis of the silicalite-1 MZM particles in Fig. 2a shows that they exhibit the same MFI-type diffraction pattern as the colloidal silicalite-1 particles. This shows that no crystalline transformation or crystal alteration occurred during the process of MZM formation.

The SEM images of the silicalite-1 MZM particles in Fig. 2b show the successful formation of well-defined micro-sized spherical particles. Consequently, the evaporation of the water within the droplets has effectively induced the assembly of the colloidal zeolite particles. The size distribution of these particles ranges approximately from 1 to 20 μm and this is an expected result due to mechanical stirring effect during the emulsion formation.

Furthermore, Fig. 2c shows some of the few observed cracked MZM particles, where it becomes clear that the colloidal silicalite-1 particles are homogenously distributed and densely packed throughout the MZM particles, confirming the efficient and complete assembly of the zeolite particles and evidencing the origin of the mesopores and the high degree of interconnectivity between them.

Fig. 2b also reveals the pores created by the interspace between the colloidal particles after the colloidal assembly, with diameters in the range of tens of nanometers in accordance with those found in Fig. 1d. For this type of colloidal assemblies, the obtained mesopore size is strongly dependent on the colloidal particle size and it becomes smaller the smaller the colloidal particle size is, as previously investigated in other colloidal assemblies.^37,55^

It can easily be shown that the aggregation of monodisperse spherical particles creates an interspace between them with a distance (pore size) centered around approximately one third of the nanoparticle diameter. This provides the possibility of tuning the mesopore size for specific applications by judicious selection of zeolite particle size.

The mesopore size distribution generated by colloidal assembly exhibits, in addition, a strong dependence on the size distribution and morphology of the colloidal particles. The colloidal particles synthesized in this work have a quasi-spherical morphology, quasi-monodisperse particle size distribution and an average particle size of 105 nm. Accordingly, the mesopore size distribution in the MZMs has an average pore size of 38 nm, which is very close to a third of the colloidal silicalite-1 nanoparticle size. The mesopore size distribution is relatively broad as expected for this type of porosity, which is expected to exhibit a trailing tail of pore sizes from the peak size down to zero due to the shrinking distance between the aggregated particles in the wedge created between them. In addition, the effect of not having a perfectly monodisperse size distribution of the colloidal particles combined with their non-spherical shape, is expected to affect the pore size distribution.

**Fig. 3:**
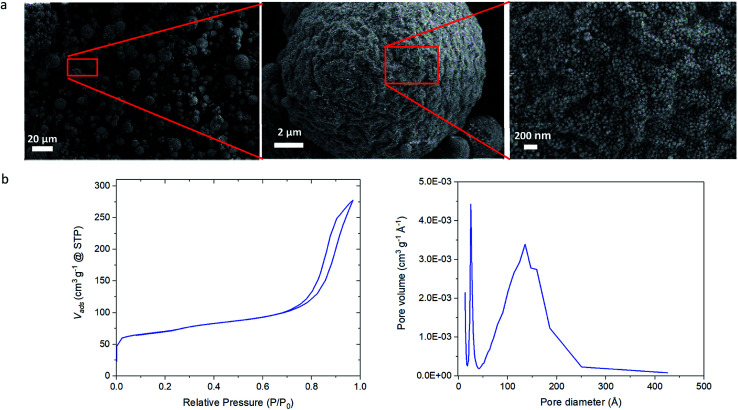
Characterization of MZSM particles. (a) SEM images of the prepared MZSMs particles. (b) N_2_ isotherm and N_2_ adsorption pore size distribution of the MZSMs.

To further extend the possibility to tailor the properties of MZMs so that multifunctional porous particles can be generated, we introduced an additional building block consisting of an amorphous silica sol mixed with the colloidal silicalite-1 sol in the aqueous phase. We mixed a 50 wt% sol of colloidal amorphous silica nanoparticles of approximately 30 nm in diameter and 50 wt% sol of colloidal silicalite-1 particles and prepared mesoporous microspheres following the procedure described in Scheme 1. Fig. 3a shows the SEM images of the resulting mesoporous zeolite/silica microsphere (MZSM) particles. The MZSMs exhibit a similar broad particle size distribution as MZM particles of approximately 1 to 20 micrometer and a quasi-spherical morphology with a wavy ridge-like surface pattern. This morphology could be attributed to the large difference between the sizes of the two building blocks and the expected less homogeneous colloidal assembly. Moreover, the different morphologies of the initial particles might contribute to this particular colloidal assembly, since the silica particles have a more spherical morphology compared to the quasi-spherical shape of the silicalite-1 particles. In addition, it is clear from Fig. 3a that the interspace between the particles is reduced in comparison with the MZMs. The porosity was further examined by N_2_ physisorption measurements (see Fig. 3b). The N_2_ isotherm shows a type IV isotherm, exhibiting the characteristics of mesoporous materials. Moreover, the isotherm shows a slightly lower slope in the hysteresis loop, indicating that the desorption of nitrogen from the pore network is more restricted than in the MZMs. In other words, the MZSM exhibits a network of smaller mesopore sizes. This is confirmed in the pore size distribution, showing that the peak mesopore size is around 14 nm. This shows that by mixing smaller silica nanoparticles with the silicalite-1 nanoparticles a significant reduction of the mesopore diameter can be achieved, since the small nanoparticles assemble in between the silicalite-1 nanoparticles as observed in Fig. 3a, partly filling the interspace between the silicalite-1 particles and therefore forming a new interspace pore distribution. Although, the morphology of the microspheres is slightly modified upon mixing two different types of nanoparticle sols, both in size and morphology, this new methodology should allow for use of mixtures of most colloidal particle sizes of the same zeolites or even mixtures of two colloidal zeolite sols of different type (*e.g.* ZSM-5, beta, zeolite A) to generate a multifunctional catalyst for particular applications requiring combinations of *e.g.* acid/base/redox catalytic activity or selectivity and/or molecular sieving effects. This ability of tuning the pore size and controlling the nature of the microspheres is likely to provide a useful means to improve the sorption and catalytic properties of such hierarchical zeolite materials.

## Conclusions

We have established an evaporation-driven colloidal assembly process for the formation of perfectly spherical mesoporous zeolite microsphere (MZM) particles. This procedure is based on a simple emulsion-based approach, where no mesopore template is used and no post-treatment conditions. Instead the large mesopores are obtained through the zeolite interparticle space generated upon the assembly and gelation of the colloidal zeolite particles. The colloidal zeolite particles are homogeneously distributed within the spherical morphology, exhibiting a well-interconnected mesoporosity with a peak size close to one third of the zeolite particle diameter. In contrast with other existing methods, the temperature and preparation time are significantly reduced and a significant increase in pore volume is obtained. The method could be further tuned by using a 50 : 50 wt% mixture of colloidal amorphous silica (30 nm in particle size) and silicalite-1 nanoparticles, directly impacting the mesopore size distribution of the microspheres as well as the pore volume and specific surface area. This new approach opens new possibilities to prepare different types of hierarchical meso-/macroporous zeolites. It enables the use of different nanoparticle sizes and the possibility of using other types of colloidal zeolite suspensions and mixtures of them. This strategy could significantly reduce the cost of production of mesoporous zeolites at an industrial scale and expands the range of available hierarchically structured materials.

## Conflicts of interest

The authors declare no conflicts of interest.

## Supplementary Material
